# Pharmacotherapy for tics in adult patients with Tourette syndrome and other tic disorders

**DOI:** 10.1007/s10072-020-04327-3

**Published:** 2020-03-13

**Authors:** James Badenoch, Andrea E. Cavanna

**Affiliations:** 1 Department of Neuropsychiatry, BSMHFT and University of Birmingham, Birmingham, UK; 2 School of Life and Health Sciences, Aston University, Birmingham, UK; 3 University College London and Institute of Neurology, London, UK; 4 Department of Neuropsychiatry, The Barberry National Centre for Mental Health, 25 Vincent Drive, Birmingham, B152FG UK

**Keywords:** Tourette syndrome, Tics, Pharmacotherapy, Guidelines, Anti-dopaminergic medications, Alpha-2 agonist medications

## Abstract

**Background:**

Tourette syndrome (TS) and persistent motor/vocal tic disorders are neurodevelopmental conditions characterised by the chronic presence of motor and/or vocal tics. Patients with TS often present with co-morbid disorders, especially attention-deficit and hyperactivity disorder (which tends to improve after childhood), and obsessive-compulsive disorder (which can persist in adulthood). We set out to explore pharmacotherapy for tics in adult patients with TS and persistent motor/vocal tic disorders, as well as its relationship with the presence of co-morbid conditions.

**Methods:**

We retrospectively reviewed the clinical characteristics and pharmacotherapy of 192 adult patients with TS (*n* = 187), persistent motor tic disorder (*n* = 3) and persistent vocal tic disorder (*n* = 2) attending a specialist clinic in the UK.

**Results:**

Anti-dopaminergic medications (*n* = 65) and alpha-2-agonists (*n* = 50) were the most commonly prescribed pharmacotherapy for tic management. A sub-group analysis revealed that co-morbid obsessive-compulsive disorder and sub-threshold obsessive-compulsive behaviours were significantly more common in patients treated with anti-dopaminergic medications than patients taking alpha-2-agonists (*p* = 0.013 and *p* = 0.047, respectively).

**Conclusions:**

The use of pharmacotherapy options for tic management observed at a specialist clinic for adults with TS reflects guideline recommendations. We found that the presence of co-morbid obsessive-compulsive disorder/behaviours correlates with the choice of anti-dopaminergic medications over alpha-2-agonists, in line with available evidence on the efficacy of anti-dopaminergic medications for the treatment of specific tic–related behavioural symptoms.

## Introduction

Tourette syndrome (TS) is a neurodevelopmental disorder characterised by the presence of both motor and vocal tics with a chronic course [1]. Individuals with chronic motor tics only fulfil diagnostic criteria for persistent motor tic disorder, whereas those with chronic vocal tics only are diagnosed with persistent vocal tic disorder. TS is frequently associated with co-morbid conditions, especially attention-deficit and hyperactivity disorder (ADHD), which tends to improve after childhood, and obsessive-compulsive disorder (OCD), which often persists in adulthood [1].

The recently published American Academy of Neurology guidelines on the assessment and treatment of tics in patients with TS and other chronic tic disorders recommend the use of alpha-2-agonists and anti-dopaminergic medications as pharmacotherapy of choice for the treatment of tics [2]. However, little is known about current practice in the use of pharmacotherapy for tic disorders in adults and how closely this practice aligns with evidence-based guidelines. In the present study, we set out to explore pharmacotherapy for tics in adult patients with TS and persistent motor/vocal tic disorders, as well as its relationship with the presence of co-morbid conditions.

## Methods

### Participants

We recruited 192 consecutive patients attending the specialist Tourette Syndrome Clinic, Department of Neuropsychiatry, National Centre for Mental Health, Birmingham, UK. All patients fulfilled current diagnostic criteria for TS or other chronic tic disorders. Patients under 16 years of age, with limited understanding of English and with severe autism spectrum disorder/learning disability were excluded from the study.

### Clinical assessment

Each patient underwent a comprehensive clinical assessment using the National Hospital Interview Schedule (NHIS) for TS [3]. The NHIS is a detailed semi-structured interview schedule covering personal and family histories, as well as demographic details. The clinical variables systematically collected for this study included age at interview, age at first tic onset, disease duration, family history of tics, psychiatric co-morbidities and pharmacotherapy. The Diagnostic Confidence Index (DCI) was used to rate the clinician’s confidence in diagnosing TS, according to the clinical characteristics of the tics and the presence of specific complex tics [4]. The DCI is a clinician-rated measure of lifetime likelihood of TS diagnosis in patients presenting with tics. The DCI score is expressed as a percentage, with higher scores indicating higher confidence that the patient has TS. Tic severity was measured using the Yale Global Tic Severity Scale (YGTSS), a clinician-rated scale assessing the severity of both motor and vocal tics across five different domains: number, frequency, intensity, complexity and interference [5, 6]. Each domain is scored 0–5 and severity scores are separately calculated for motor and vocal tics by summing together the individual domain scores. Finally, the combined motor and vocal tic severity scores produce a total tic severity score. An additional measure of overall impairment is scored 0–50. These two scores are combined to produce the total YGTSS score, ranging from 0 to 100, with higher scores indicating increased tic severity.

### Statistical analysis

All statistical analyses were conducted using parametric testing on continuous variables that were normally distributed and non-parametric testing on continuous variables that were not normally distributed. The chi-square test was used for non-continuous variables.

## Results

Out of the 192 adult patients in our clinical sample, 187 had a diagnosis of TS, 3 persistent motor tic disorder and 2 persistent vocal tic disorder. The demographic and clinical characteristics of the sample are shown in Table 1.

**Table 1:** Demographic and clinical characteristics of the sample of patients with Tourette syndrome and other chronic tic disorders (*n* = 192)

Male gender (*n*, %)	134 (69.8)
Age (median, range)	24 (16–68)
Age at first tic onset (mean, SD)	8.4 (4.1)
Family history of tics (*n*, %)	101 (52.6)
OCD (*n*, %)	49 (25.5)
OCB (*n*, %)	130 (67.7)
ADHD (*n*, %)	56 (29.2)
ASD (Asperger syndrome) (*n*, %)	23 (12.0)
Mild LD (*n*, %)	14 (7.3)
Depression (*n*, %)	71 (37.0)
Anxiety (*n*, %)	34 (17.7)
DCI (mean, SD)	70.2 (18.3)
YGTSS tic severity score (mean, SD)	29.3 (8.3)
YGTSS impairment score (mean, SD)	26.0 (8.6)
YGTSS total score (mean, SD)	55.4 (14.6)

Abbreviations. *OCD*, obsessive-compulsive disorder; *OCB*, obsessive-compulsive behaviours; *ADHD*, attention-deficit and hyperactivity disorder; *ASD*, autism spectrum disorder; *LD*, learning difficulty; *DCI*, Diagnostic Confidence Index; *YGTSS*, Yale Global Tic Severity Scale

About two thirds of the patients (66.7%) were on some form of pharmacotherapy for their tics (Table 2). Anti-dopaminergic medications (*n* = 65) and alpha-2-agonists (*n* = 50) were the most commonly prescribed pharmacological classes. All patients taking alpha-2 agonists were prescribed clonidine, the most commonly used tic-suppressing medication.

**Table 2:** Pharmacotherapy in the sample of patients with Tourette syndrome and other chronic tic disorders (*n* = 192)

Any medication for tics	128 (66.7)
Alpha-2 agonists plus anti-dopaminergic medications	10 (5.2)
Alpha-2 agonists	50 (26.0)
Anti-dopaminergic medications	65 (33.9)
First-generation anti-dopaminergic medications	14 (7.3)
Haloperidol	7 (3.7)
Pimozide	2 (1.0)
Other	5 (2.6)
Second-generation anti-dopaminergic medications	51 (26.6)
Aripiprazole	37 (19.3)
Risperidone	11 (5.7)
Other	3 (1.6)
Topiramate	13 (6.8)

A sub-group analysis compared the clinical characteristics of patients taking anti-dopaminergic medications only (*n* = 55) with those of patients taking alpha-2-agonists only (*n* = 40). The only significant difference between the two sub-groups was identified when analysing the presence of co-morbidities: patients with OCD were more likely to be taking anti-dopaminergic medications than alpha-2-agonists (77.8% versus 22.2%, chi-square = 6.117, *p* = 0.013). The same difference was observed with sub-threshold obsessive-compulsive behaviours (65.1% versus 34.9%, chi-square = 3.960, *p* = 0.047).

## Discussion

The use of pharmacotherapy for tic management observed in a large and representative clinical sample of adult patients with TS and other chronic tic disorders reflects current guideline recommendations [2]. The DCI scores in our sample are in line with the scores reported by patients seen at specialist clinics for the treatment of tic disorders, indicating high diagnostic confidence [4]. Our findings are also in line with the results of a recent study on pharmacological prescriptions in Canadian children with tic disorders [7]: the growing use of clonidine and aripiprazole appears to be driven by emerging evidence supporting their efficacy on tics, as well as their safety [8, 9]. Interestingly, recommendations for first-generation anti-dopaminergic agents such as haloperidol and pimozide were comparatively limited, likely due to the greater adverse effects (especially sedation, weight gain, hyperprolactinaemia and extrapyramidal symptoms) associated with these medications [9]. Likewise, the wider use of risperidone compared with pimozide reflected the recent recommendations about its different tolerability pattern, wider spectrum of action and potentially higher certainty in the effect estimate [9].

We found that the presence of co-morbid OCD/obsessive-compulsive behaviours correlates with the choice of anti-dopaminergic medications over alpha-2-agonists, in line with available evidence on the efficacy of anti-dopaminergic medications for the treatment of specific tic–related behavioural symptoms. In a recent study on adults with TS, aripiprazole significantly improved OCD and showed a trend toward improvement of other behavioural co-morbidities [10]. In the same study, patients with TS and co-morbid OCD tended to decide in favour of pharmacotherapy, suggesting that the decision-making process for or against medical treatment could be influenced by the presence of specific behavioural co-morbidities. Moreover, there is some evidence that patients with both TS and tic-related OCD do not respond as well to serotonergic medication (first-line pharmacotherapy for OCD) compared with patients with primary OCD only [2]. It can be hypothesised that the beneficial effects of aripiprazole on both tics and tic-related OCD are related to this medication’s adaptive pharmacological profile, which exhibits an influence on multiple neurotransmitter systems in addition to dopaminergic pathways, as well as to shared mechanisms underpinning the expression of both tics and tic-related obsessive-compulsive behaviours.

The main limitations of the present study include its retrospective approach and referral bias, as participants were recruited from a tertiary referral centre and may therefore not be representative of the wider community. Future research is needed to fully understand how the phenotypic heterogeneity of patients with TS and other chronic tic disorders affects pharmacotherapy across the lifespan.
